# Video-Assisted Breast Surgery (VABS) and Vacuum-Assisted Breast Biopsy (VABB) for Fibroadenoma Mammae on Patients’ Satisfaction: A Preliminary Study

**DOI:** 10.31557/APJCP.2021.22.11.3615

**Published:** 2021-11

**Authors:** Kristanto Yuli Yarso, Muhammad David Perdana Putra, Monica Bellynda, Akhmad Azmiardi

**Affiliations:** 1 *Department of Surgery, Division of Oncology, Faculty of Medicine, Universitas Sebelas Maret, Surakarta, Indonesia. *; 2 *Department of Surgery, Faculty of Medicine, Universitas Sebelas Maret, Surakarta, Indonesia. *; 3 *Department of Public Health, Faculty of Public Health, Universitas Bangun Nusantra, Sukoharjo, Indonesia. *

**Keywords:** Video-assisted breast surgery, vacuum-assisted breast biopsy, minimally invasive breast surgery

## Abstract

**Objective:**

This study aimed to compare the patients’ satisfaction level after fibroadenoma surgery with Video-Assisted Breast Surgery (VABS) and Vacuum-Assisted Breast Biopsy (VABB) techniques.

**Methods:**

Patients who underwent VABS or VABB for a diagnosis of fibroadenoma mammae at the Oncology Clinic in Solo, Indonesia were included in this study. Clinical and demographic data were obtained from medical records. Direct or telephone interviews were performed and the patients were asked to complete Universitas Sebelas Maret Breast Satisfaction Questionnaire 8 (UNS-BsQ8) questionnaire.

**Results:**

A total sample of 16 patients with VABS and 26 patients with VABB were recruited. All the patients were confirmed to have fibroadenoma based on the pathological result. The mean total scores for VABS and VABB were 34.50 ± 2.094 and 31.57 ± 3.081, respectively (P= 0.137). Out of 8 questions, only 3 items had statistically significant differences. VABS had higher mean score than VABB in terms of surgery cost (P = 0.002), pain in surgery site (P = 0.006), and pain in shoulder (P = 0.013).

**Conclusion:**

There was no significant difference in terms of overall patients’ satisfaction level between both groups. However, VABS had a higher mean score than VABB in terms of cost and pain.

## Introduction

Fibroadenoma (FAM) is a benign tumor of the breast in adolescents. Fibroadenoma is also the most diagnosed breast tumor (Lee and Soltanian, 2015). It accounts for 69% of all breast masses and 44-94% of all biopsied breast tumors. Minimal invasive techniques in fibroadenoma including endoscopic surgery, vacuum-assisted percutaneous biopsy, and ablative procedure can be used to remove the lump up to 3 cm in size (Lee and Soltanian, 2015).

Since 1992, video-assisted breast surgery (VABS) for the breast has been developed mainly in the field of plastic surgery (Tamaki et al., 2002). Today, partial or total breast endoscopic surgery can be performed to treat benign breast tumors to improve the cosmetic outcome (Lakoma and Kim, 2014). Besides, VABS has been extensively used and has been accepted as an alternative and less invasive approach to open breast surgery more and over. However, minimal access and endoscopic breast surgery have been used commonly in clinical practice nowadays. VABS for breast cancer surgery, even for Sentinel node biopsy was described in 2008 by Yamasita. In 1998, Kitamura reported the first use of endoscopic surgery for removal of benign breast tumors in six patients, and in 2001 he reported a more extensive experience in 36 patients with benign breast lesions (Kitamura et al., 1998; Yamashita and Shimizu, 2008; Hong and Shin, 2010).

On the other hand, complications are more frequently observed following VABS. This includes skin bruising, blistering, nipple-areolar complex necrosis, and muscle flap necrosis in skin-sparing mastectomy with reconstruction. It may be associated with greater intra-operative blood loss than open breast surgery. These complications were found in mastectomy for breast cancer using VABS (Kitamura et al., 2002; Zagouri et al., 2008; Fan et al., 2009; Domeyer et al., 2010).

Besides VABS, vacuum-assisted breast biopsy (VABB) is another minimally invasive surgical technique used to treat FAM. VABB was first developed in 1995 by radiologist Fred Burbank in California in association with Mark Retchard. VABB has gained popularity because of its minimally invasive procedure. Thus, it is gentler to the patient and can be done with local anesthesia. It is also potentially more cost-effective by avoiding hospitalization. It can obtain a larger amount of tissues with contiguous specimens with the possibility of acquiring multiple specimens with single needle insertion (Lui and Lam, 2010; Eller et al., 2014; Bennett and Saboo, 2019).

One of the considerations in choosing a surgical method is the patient’s satisfaction level. The satisfaction level in measuring devices that can influence an agency’s health service quality, such as patient satisfaction and patient dissatisfaction, should be assessed for every patient. It can then improve the quality of services provided to patients to be achieved according to patient’s wishes (Naomi, 2005).

Therefore, this study aims to compare the patients’ satisfaction level after fibroadenoma surgery with VABS and VABB techniques.

## Materials and Methods

*VABB*

The patient was referred for ultrasonography (US) and fine needle aspiration (FNA) examinations the day after visiting the oncology clinic. The patients with the US and FNA concluding benign were then offered surgery to remove the tumor. The procedures for VABB and VABS were explained. The procedure was done according to the patients’ preference.

Local anesthesia was performed for single and small tumors. Meanwhile, general anesthesia was needed for big and multiple tumors. An 8G needle was used. It was inserted through the periareolar or inframammary line depending on tumor location. Local anesthesia with lidocaine adrenaline was injected around the tumor with an 18g needle with US guiding before the procedure starts. The tumor was operated using an active vacuum to pull the biopsy tissue into the sampling notch; an inner cutter was then driven in to cut through breast tissue. The biopsy specimen was then drawn outside the breast into a specimen collection chamber by vacuum. Contiguous samples were obtained by rotating the needle with the aperture position, pointing towards a different clock position. Suction was then applied again to capture another specimen. In this way, multiple specimens can be retrieved without the need for needle re-insertion (Park and Hong, 2014; Papathemelis et al., 2017). VABB can be employed to remove any size of FAM, but 6.13 cm was the greatest size of fibroadenoma ever reported to be completely removed using this method. This giant fibroadenoma has been taken in 458 slices (Bellynda and Yarso, 2021).

In this study, the tumor removal under ultrasound guiding was performed. The researchers counted the number of tissues removed from every tumor. In all cases, 6-inch elastic bandages were applied after the procedure.

*VABS*

**Figure 1:**
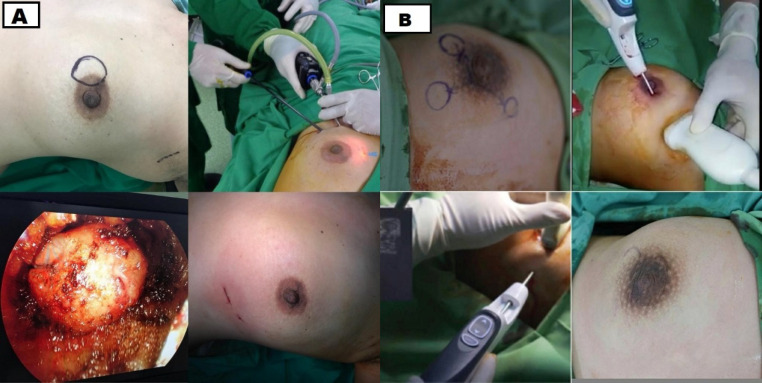
A, VABB operating procedure using gas and a 5mm camera with the approach of the inframammary fold; B, VABB procedure using an 8G needle with areolar approach

Some patients choose VABS due to its minimal scar result. For VABS with gas, it usually has three incisions. One for the camera and another two for the surgical instrument. 0o and 30o 10-mm cameras are usually used. For small tumors, a 5-mm camera can be utilized. The tumor was removed through an incision from where the camera was inserted. Large diameter tumors need to be cut into pieces before they are completely removed. For the instrument, an incision is made about 5 cm next to the camera entry. As for the gasless procedure, the researchers only used Hoek and incision 2-3 cm long from the hidden place. All instruments were entered from the same incision location (Figure 1).

There are two incisions for the surgical approach. An incision located in the lateral breast up to the axillary line is used to approach tumor that is located in the upper quadrant. Another incision is placed along the inframammary fold for tumors located in the lower breast quadrant. This is to avoid injury to the primary lactiferous ducts below the nipple. The patient is in supine position. The arm was abducted at 90° to avoid disturbing the surgical and instrument maneuver. Usually, video monitors are set on the opposite the operator and assistants to watch the procedure inside the breast. This study’s technique was based on the technique described in previous publications (Nakajima et al., 2002; Lee et al., 2006; Yamashita and Shimizu, 2006).

*Patient Satisfaction*

Satisfaction is a feeling of pleasure someone obtained from an action or in accordance with the expected results. Satisfaction assessment is an interaction between expectations and results obtained after using the services provided (Naomi, 2005). It can affect the doctor’s services provided to patients, especially in the services’ quality obtained by patients after the treatment given (Dahlui and Aziz, 2012).

Assessment could be taken from the level of patient satisfaction in the presentation very satisfied, satisfied, and not satisfied. Assessing each level of patient satisfaction from these results can improve the quality of services provided for patients to be achieved according to patient’s wishes. To conduct an analysis of patient satisfaction with breast surgery, one of the questionnaires used to evaluate benign breast tumor surgery satisfaction in the institution is Universitas Sebelas Maret Breast Satisfaction Questionnaire (UNS BsQ8). This questionnaire was developed in our academic center. Validity and reliability test had been done previously and it is eligible to use for assessing patients’ satisfaction level after fibroadenoma surgery. There are numerous types of questionnaire to assess the quality of life of breast cancer patients, such as RAND SF-36 that had been validated in Indonesian version (Ramadhanty et al., 2019). However, there is no questionnaire yet to assess fibroadenoma patient’s satisfaction level after surgery (Putra et al., 2020).

*Patients and Methods*

This descriptive analytic study used a cohort retrospective design to compare the patients’ satisfaction who had performed VABS and VABB. With the starting point when undergoing the procedure and the endpoint when the patients checked themselves three months later. The study was conducted at Kasih Ibu and Indriati Hospital. Data collection was carried out from January 1st – 31st 2020. Purposive sampling was done in which the researchers relied on their judgment when choosing a member of the population to participate in this study.

The inclusion criteria was patients with FAM pathology report. The exclusion criteria were patient with malignant breast tumor; refused the procedure; and less than three months after the surgery. Ethical clearance had been submitted and approved by the medical ethics committee of dr. Moewardi Hospital No:1.073/X/HREC/2020.

*Questionnaire*

To measure patients’ satisfaction level after VABS or VABB surgery, UNS-BsQ8 questionnaire was used. The questionnaire question list is described in Table 1. The questionnaire consists of eight items. The questionnaire was also designed a combination of ordinal scaled (grading scale from 1–poor to 5–excellent). Final scoring was carried out by summing up the individual item scores to produce a range of 8 to 40. with higher scores representing greater satisfaction (Putra et al., 2020). In this study, we did minor modification on question number 3 from ”The cost of surgery is expensive” to ” The cost of surgery is not expensive” by adding the world “not” to adjust that practically, patients who were satisfied have the tendency to agree that the cost is not that expensive to get a satisfying result. Score 5 in this question means that the subject really agree that the procedure is not expensive.

The patients were asked to complete the questionnaire online. Patients who did not respond within one month were interviewed by phone. The collected data were analyzed using IBM SPSS 25.0. P-value <0.05 was considered to be statistically significant.

## Results

Based on the medical records from 2017 to 2019, 49 patients with benign breast tumor underwent VABS or VABB. All the subjects were interviewed in January 2020. Seven patients refused to be interviewed, resulting in 16 VABS subjects and 26 VABB subjects. 

Other variables that might affect satisfaction, such as age, marital status, education level, tumor size and number, and complication were also obtained. Patients’ characteristic analysis is described in Table 2. All variables were divided into two groups. There was no significant difference between two groups in all variables, which means that these confounding did not affect the patients’ satisfactory level.

**Figure 2:**
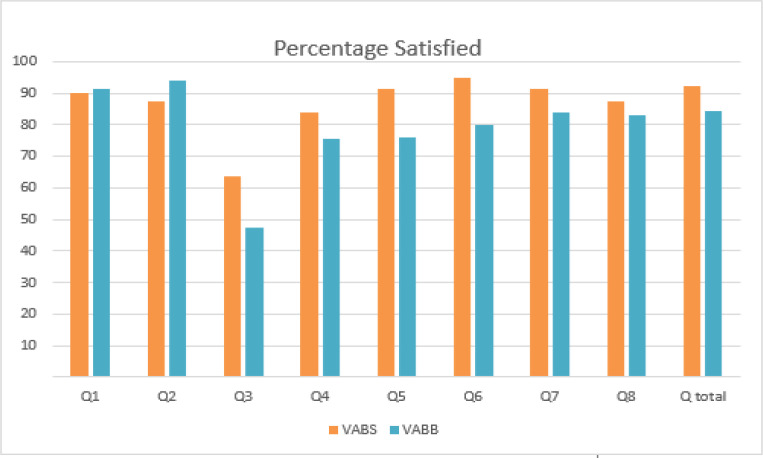
The Proportion of FAM Patients who were Satisfied with VBS and VABB Procedure

The total score of eight questions was compared between VABS and VABB groups (Table 3 and 4). The mean total value between two groups was 34.50 ± 2.09 in VABS and 31.57 ± 3.08 in VABB. The result was not significant (P = 0.137). The proportion of patients who were satisfied with VABS and VABB procedure is illustrated in Figure 2. 

Each question was also compared between two groups. No significant statistical difference was found in question 1, 2, 4, 7, and 8. In Question 1 (Q1), the mean score for VABS was 4.50 ± 0.73 and VABB was 4.58 ± 0.643 (P = 0.783), and Question 2 (Q2) with mean score for VABS was 4.38 ± 0.719 and VABB was 4.69 ± 0.549 (p=0.117). Although not statistically significant, VABB had a higher mean score than VABS in general condition and wound healing progress after surgery. In opposite, VABS had a higher mean score in question 4 (Q4) with a mean score of 4.19 ± 0.750 for VABS and 3.77 ± 0.765 for VABB (P = 0.100); question 7 (Q7) with a mean score of 4.56 ± 0.727 for VABS and 4.19 ± 0.634 for VABB (P = 0.051); and question 8 (Q8) with a mean score of 4.38 ± 0.885 for VABS and 4.15 ± 0.967 for VABB (P = 0.428).

Cost of surgery, pain in the surgical site, and pain in the shoulder show a statistically significant mean score difference between VABS and VABB group. In question 3 (Q3), VABS group had a mean score of 3.18 ± 0.834 while VABB group had a mean score of 2.38 ± 0.697 (P = 0.002). Another significant difference was also seen in question 5 (Q5) and question 6 (Q6). Q5 assesses the pain in the location of surgery, resulting in a higher mean score in VABS group (4.56 ± 0.727) compared to VABB group (3.81 ± 0.895) with p = 0.006. In Q6, which assesses pain in the shoulder, most patients did not complain any significant symptoms. Again, VABS group has higher mean score value (4.75 ± 0.577) than VABB group (4.00 ± 1.131) with P = 0.013. 

The complications after the surgery were mainly ecchymosis that disappeared in 10-14 days without any hospitalization. There was also no infectious complication following the surgery. Only one patient complained of lysis of old hematoma in the second week from the VABB group. 

**Table 1:** UNS-BsQ8 Questionnaire

Question	Description	Score
1	How Would you classify your condition after surgery?	1: poor 2: fair
2	How would you classify your wound healing progress?	3: good 4: very good 5: excellent
3	The cost of surgery is not expensive	1: strongly disagree 2: disagree
4	There is no change of your breast shape	3: neutral 4: agree 5: strongly agree
5	How frequent do you feel pain in the wound surgical site?	1: always 2: often
6	How frequent do you feel pain in the shoulder?	3: neutral 4: seldom 5: never
7	How would you classify the appearance your scar after surgery?	1: poor 2: fair 3: good 4: very good 5: excellent
8	Scar after surgery makes me uncomfortable	1: always 2: often 3: neutral 4: seldom 5: never

**Table 2:** Characteristic and Confounding Factors of Patients in This Study

Variable		Total	VABS N (%)	VABB N (%)	P value
Age	< 35 years old	28 (66.7)	10 (62.5)	18 (69.2)	0.453
	>= 35 years old	14 (33.3)	6 (37.5)	8 (30.8)	
Educational	High school	12 (28.6)	4 (25.0)	8 (30.8)	0.478
	Bachelor	30 (71.4)	12 (75.0)	18 (69.2)	
Marital	Unmarried	13 (31.0)	4 (25.0)	9 (34.6)	0.356
	Married	29 (69.0)	12 (75.0)	17 (65.4)	
Occupation	Unemployed	13 (31.0)	7 (43.8)	6 (23.1)	0.110
	Employee	29 (69.0)	7 (56.2)	20 (76.9)	
Tumor Size	<= 2 cm	25 (59.5)	8 (50.0)	17 (65.4)	0.225
	> 2 cm	17 (40.5)	8 (50.0)	9 (34.6)	
Tumor Number	<=2	34 (81.0)	13 (81.3)	21 (80.8)	0.673
	>2	8 (19.0)	3 (18.8)	5 (19.2)	
Complication	Negative	28 (66.7)	12 (70.0)	16 (62.0)	0.417
	Positive	14 (33.3)	4 (30.0)	10 (38.0)	

**Table 3:** Comparison of the Distribution of Satisfaction Level in VABS and VABB Group Based on 8 Questions in UNS-BsQ8 Questionnaire

Question	VABS	VABB	VABS	VABB	VABS	VABB	VABS	VABB	VABS	VABB
	N (%)	N (%)	N (%)	N (%)	N (%)	N (%)	N (%)	N (%)	N (%)	N (%)
	Score 1		Score 2		Score 3		Score 4		Score 5	
1	0 (0)	0 (0)	0 (0)	0 (0)	2 (12.5)	2 (7.7)	4 (25.0)	7 (26.9)	10 (62.5)	17 (65.4)
2	0 (0)	0 (0)	0 (0)	0 (0)	2 (12.5)	1 (3.8)	6 (37.5)	6 (23.1)	8 (50.0)	19 (73.1)
3	0 (0)	2 (7.7)	2 (12.5)	13 (50.0)	11 (68.8)	10 (38.5)	1 (6.3)	1 (3.8)	2 (12.5)	0 (0)
4	0 (0)	0 (0)	0 (0)	0 (0)	3 (18.8)	5 (19.2)	7 (43.8)	16 (61.5)	6 (37.5)	3 (11.5)
5	0 (0)	0 (0)	0 (0)	2 (7.7)	2 (12.5)	7 (26.9)	3 (18.8)	11 (42.3)	11 (68.8)	6 (23.1)
6	0 (0)	0 (0)	0 (0)	5 (19.2)	1 (6.3)	1 (3.8)	2 (12.5)	9 (34.6)	13 (81.3)	11 (42.3)
7	0 (0)	0 (0)	0 (0)	0 (0)	2 (12.5)	3 (11.5)	3 (18.8)	15 (57.7)	11 (68.8)	8 (30.8)
8	0 (0)	0 (0)	0 (0)	2 (7.7)	4 (25.0)	4 (15.4)	2 (12.5)	8 (30.8)	10 (62.5)	12 (46.2)

**Table 4:** Analysis and Description of Difference in 8 item Questionnaire of Patients Satisfaction Regarding VABS vs VABB

Question	VABS					VABB					U-value	P-value
	N	Mean	SD	Min	Max	N	Mean	SD	Min	Max		
Q1	16	4.50	0.730	3	5	26	4.58	0.643	3	5	199	0.783
Q2	16	4.38	0.719	3	5	26	4.69	0.549	3	5	157	0.117
Q3	16	3.18	0.834	2	5	26	2.38	0.697	1	4	101.5	0.002
Q4	16	4.19	0.750	3	5	26	3.77	0.765	2	5	150.5	0.100
Q5	16	4.56	0.727	3	5	26	3.81	0.895	2	5	108.5	0.006
Q6	16	4.75	0.577	3	5	26	4.00	1.131	2	5	123	0.013
Q7	16	4.56	0.727	3	5	26	4.19	0.634	3	5	139.5	0.051
Q8	16	4.38	0.885	3	5	26	4.15	0.967	2	5	180	0.428
Q total	16	34.5	2.094	30	40	26	31.57	3.081	28	40	151	0.137

## Discussion

From the result of this study, it can be concluded that there was no difference in overall satisfaction between VABB and VABS group. It was shown by the mean total value in both groups, which was not significant. Although not statistically significant, VABS group had a higher total mean score of 34.50 ± 2.094 while VABB group scored 31.57 ± 3.081.

Among eight question, only three had statistically significant differences, which were Q3, Q5, and Q6. Those three questions assess the cost of surgery (P = 0.002), pain in surgical site (P = 0.006), and pain in the shoulder (P = 0.013). VABS group had a higher mean score than VABB group.

Currently, no studies have already compared patients’ satisfaction level among these two minimally invasive techniques in FAM patients. Patients with middle and upper monthly income tend to choose VABB surgery because it has better cosmetic results with only small incisions than conservative surgery, even though this procedure is a lot more expensive. The cost of VABB surgery is 500 USD more expensive compared to VABS surgery. Q3 showed that the satisfaction score was higher in VABS group in terms of cost for surgery. It is understandable because of the difference in costs arising from the consumables probe. 

Moreover, a woman’s dissatisfaction usually lies in aesthetics. Women have concerns about the cosmetic outcomes of benign breast tumor resections (Lui and Lam, 2010; Soybir and Fukuma, 2015). Minimally invasive techniques appear to be well tolerated and relatively safe techniques. Complications commonly observed following open breast surgery are seroma (hydrops) formations, hematomas, infections, and prosthesis-related complications. Complications after mastectomy are more frequently observed. These include skin bruising and blistering from electrocautery, skin and muscle flap necrosis, and necrosis of the nipple-areolar complex. These complications are less frequent following VABS procedure. (Kitamura et al., 2002). 

It is possible that complications may affect the satisfaction level. It might be also resulted from some limitations such as lack of skill and training, and also limitations of the endoscopic instruments (Gverić et al., 2011; Wu et al., 2017). In this study, there were no patients who felt unsatisfied after VABS procedure. Complications after procedure in this study, such as ecchymosis and hematoma, were not significantly different in both groups and did not interfere with patient satisfaction in assessing the outcome of this procedure.

Aside from VABS, current consensus indicates and recommends the use of ultrasound-guided VABB (Eller et al., 2014). Ultrasound-guided VABB is a suitable and approved method for the complete excision of benign and symptomatic breast lesions. This procedure also represents an alternative to open excision. Ultrasound-guided VABB, if done properly, is almost painless. VABB is a safe method of breast biopsy and removing benign breast nodules. It is also associated with less complications and only result in mild pain during and after the procedure. It has also been approved by FDA (Food Drug Administration) in the United States of America and The National Institute for Health and Care Excellence (NICE) in the United Kingdom as a use for the treatment of benign breast tumors (Eller et al., 2014; Park and Hong, 2014; Papathemelis et al., 2017).

Numerous studies have also tested that VABB is less invasive, causes less damage, speeds time between detection and diagnosis, and costs less than open surgery. While much progress has been made, there are still rooms for improvement for developing new technologies that can increase accuracy, safety, and cost-effectiveness (Park and Hong, 2014; Papathemelis et al., 2017).

The limitation of this study is that the number of participants is not that much. This is due to the cost that is expensive and patients’ preference to choose conservative surgery that is cheaper and can be covered by the national health insurance.

In conclusion, there is no difference in patients’ satisfaction level between VABS and VABB groups. However, VABS group was significantly superior than VABB group in terms of pain and the cost of surgery. 

## Author Contribution Statement

Kristanto Yuli Yarso: author, corresponding author, data, method. David Perdana Putra: data, analysis, writing. Monica Bellynda: editing, writing, proofreading. Ahmad Azmiardi: statistic.

## Funding Statement

This research did not receive any specific grant from funding agencies in the public, commercial, or not-for-profit sectors.
